# Social Engagement in the Fight Against COVID-19 in the Urban and Peri-Urban Areas of Cotonou (Benin, Sub-saharan Africa): Acceptability of the Vaccination and Tracking Program

**DOI:** 10.3389/fmed.2022.857890

**Published:** 2022-06-03

**Authors:** Alessia Maccaro, Davide Piaggio, Iyabosola Busola Oronti, Marius Vignigbé, Antoinette Gbokli, Roch Houngnihin, Leandro Pecchia

**Affiliations:** ^1^Applied Biomedical Signal Processing and Intelligent e-Health Lab, School of Engineering, University of Warwick, Coventry, United Kingdom; ^2^Laboratoire d'Antropologie Médicale Appliqué, University of Abomey Calavi, Cotonou, Benin; ^3^School of Engineering, Università Campus Bio-Medico di Roma, Via Álvaro del Portillo, Roma, Italy

**Keywords:** COVID-19, vaccine, acceptance, tracking, social engagement, pandemic management, Benin, Sub-Saharan Africa

## Abstract

**Introduction:**

This article aims at investigating social engagement in the fight against the COVID-19 pandemic in low-resource settings (LRSs). In particular, it focuses on Benin (Sub-Saharan Africa), and reports the results of a field study that investigated the local people's acceptance of the vaccine and the tracking program.

**Methods:**

This project is the product of a collaboration between the ABSPIE (Applied Biomedical and Signal Processing E-Health) Lab of the University of Warwick (UK) and the LAMA (Laboratoire d'Antropologie Medical Appliqué) of the University of Abomey Calavi (Benin). This international multidisciplinary collaboration brought together engineers, sociologists, anthropologists, and bioethicists. In light of the aims of the project, a qualitative methodology was deemed appropriate. The research team prepared two questionnaires that provided the basis for semi-structured interviews that took place between June and August 2021.

**Results:**

The research team interviewed 34 Beninese respondents, comprising people aged 60+ (with multiple comorbidities), who were primarily healthcare workers and/or traditional therapists. The results of this work highlight the fact that there is widespread reticence about the vaccination program in Benin, both due to local beliefs and uncertainty about governmental management. In this study, we uncovered several local reasons interfering with the involvement of the population in the vaccination campaign against COVID-19, e.g., the existence of traditional medical practices considered as valid alternatives to vaccines, and many beliefs showing a fear of neo-colonialism hidden in the pandemic threat. Yet, another hindrance can be traced to shortcomings in the management of the vaccination campaign which resulted in obstacles to the implementation of the program.

**Conclusions:**

This work does not intend to denounce any governmental effort or foster a regressive mindset, but shows how the overall confusion (defined by the World Health Organization as infodemic) linked to the pandemic and its management has caused even more dramatic consequences in LRSs. In addition, the paper proposes a specific framework for the interpretation and management of bioethical and biomedical issues in LRSs that the authors are validating in their current research.

## Introduction

The COVID-19 pandemic constitutes the most important public health challenge of our times. Globally, for the past 2 years, health systems have been facing a great strain due to the lack of means and resources [e.g., priority medical devices (MDs), intensive care unit (ICU) beds, COVID-19 tests and vaccines], as well as personnel and specialized knowledge. Consequently, even high income countries experienced a situation typical of LRSs (1–3). As of January 2022, the world experienced the third wave of the COVID-19 healthcare crisis, which was heavily influenced by the appearance of five variants of the SARS-COV-2 virus (4, 5). The most effective solution identified was mass vaccination. For this reason, many companies promptly started working on vaccine development and testing. The genetic sequence of SARS-COV-2 was published on January 1^1^, 2020 and the first COVID-19 vaccine candidate entered human clinical testing with an unprecedented celerity on 16 March 2020.

To this end, the scientific community joined forces to develop the most promising vaccine candidates. Out of several vaccines developed on different technology platforms (6), only seven obtained the authorization for human administration (see footnote 1). Currently, more than 9 billion vaccine doses have been administered^2^ worldwide. However, a great gap in coverage still exists between countries such that while some are already administering the third or fourth dose (e.g., more than 60% fully vaccinated persons in the US), others are barely covered by the first dose^3^. Specifically, in high-income and upper-middle income countries respectively, 71 and 73% of the population completed the vaccination cycle (as of January 17, 2022). Contrarily, in low- and lower-middle income countries respectively, only 4.9 and 36% of the population received the vaccine. Overall, only 50.15% of the population completed the vaccination cycle^4^.

Unfortunately, inequalities and differences among countries are exacerbated by the cost of vaccines. However, cost is evidently not the only reason why vaccine deployment is unequitable among different countries. Other reasons include governmental preparedness, poor transport links and inability to maintain a cold chain, which is vital for preserving some vaccines. This situation resulted in governments, scientific institutions, and healthcare professionals embarking on unprecedented collaborations to promote equitable access to COVID-19 vaccines through speedy deployment and distribution in low- and middle-income countries (LMICs). This materialized in the COVAX initiative, co-led by the Gavi Alliance, the Coalition for Innovations in Epidemic Preparedness (CEPI) and the WHO^5^ The vaccine distribution strategy references an ethical framework established by the WHO's Strategic Advisory Group of Experts' on Immunization (SAGE) (7–9). Nevertheless, these efforts did not automatically translate into popular support for COVID-19 vaccines worldwide (10), and particularly in LMICs (11, 12).

One of the main reasons for hesitation in relation to vaccination is the lack of clear information from the government and scientific institutions, exacerbated by the circulation of inaccurate news through social media and the internet (13). This phenomenon, referred to by the WHO as “infodemics”^6^ (14– 16) or “disinfodemic” (17), has particular repercussions in LMICs, where it feeds widespread negationism (18). Despite the COVAX target to provide up to 600 million COVID-19 doses by the end of 2021, and the fact that 52 African countries received almost 177 million doses (19) of vaccines as of January 2022, the vaccination target for Africa was not reached. The authors of this paper believe that this may not be linked to a lack of supply chain, but rather, to the local culture and pessimistic approach relative to the vaccines.

This study was limited to Benin since it is inhabited by a melting pot of ethnic groups, is considered the “cradle” of the Vodun religion and an exemplar for the traditional culture dominant in Sub-Saharan Africa, and is also enrolled in the COVAX initiative that saw the deployment of a specific National Vaccination Plan^7^,^8^. A total of 26,036 confirmed COVID-19 cases with 162 deaths were reported to the WHO in Benin from January 3, 2020 to January 13, 2022, while no less than 1,897,214 vaccine doses had been administered as of January 3, 2022. This means that 11% of the population completed the vaccination cycle, and 15% received at least one dose^9^. Interestingly, according to available literature (20, 21), Benin is one of the countries with the lowest COVID-19 vaccine acceptance rate. In general, the reported data related to the spread of COVID-19 in Africa are strictly dependent on tracking systems, which are evidently problematic in LMICs due to institutional and technical under-preparedness (22, 23). Therefore, an in-depth analysis of the social reasons for these phenomena is essential to understanding the underlying problems in order to favor a better vaccine rollout and acceptance. It is important to note that the COVID-19 vaccine was introduced pari passu with the rich tradition of healthcare treatments that offered alternative herbal-based therapies (EB + anti-COVID-19, Api-COVID-19, etc.), which were well received by the population (24–26).

This work presents the results of a multidisciplinary collaboration, which focused on the analysis and critical appraisal of the perceptions of the Beninese population and the novel challenges brought about by the COVID-19 pandemic. It relies on a previously created *hermeneutic heuristic framework* that combines the theoretical structure of intercultural bioethics and the empirical, inductive and contextualized approach (by design) of the sciences (in this case biomedical engineering), thus proposing solutions inspired by the concept of “frugality” that takes account of the particularism of each context, while tending toward a model of universalism. Part of this framework relating to the frugal design of medical devices is already published (27), while the other part is currently under consideration. Our research goals included capturing popular perceptions about COVID-19 and strategies put in place for fighting the pandemic (i.e., test and trace, vaccines), investigating the main reasons behind the emerging local perspectives, highlighting the main local reactions, and understanding the differences between the perspectives and reactions of the inhabitants of urban and peri-urban areas. Given this background, the results of the study will be a proxy to understanding the overall level of commitment in the fight against COVID-19, and to support community engagement for the future of public health management in LMICs.

## Materials and Methods

This article is the outcome of an international multidisciplinary collaboration between the ABSPIE Lab of the University of Warwick (UK) and the LAMA of the University of Abomey Calavi (Benin) that brought together engineers, sociologists, anthropologists and bioethicists, with the common goal of bringing key interdisciplinary issues that affect LRSs to the attention of the world. The project was divided into three phases, mainly; (a) the hermeneutical (interpretative) phase—conducted as a field study by the researchers of the University of Abomey-Calavi, (b) the analytical phase, and (c) the synthetical phase. The last two phases were led by researchers at the University of Warwick.

In light of the aims of the project, a qualitative methodology and inductive approach were deemed appropriate and selected for the first phase. The research methodology was structured around data collection methods (semi-structured interviews and focus groups), with experts drawn from groups specified in the criteria contained in the government's vaccination strategy as dictated by the COVAX initiative, i.e., the healthcare sector, traditional local medicine practitioners (*traditherapeutes*), as well as representatives of the local people, in particular, carriers of comorbidities, people aged 60 and above, and vaccinated subjects. The research team prepared the layout of two questionnaires (attached in the Supplementary Materials), which formed the basis for semi-structured interviews (held either in French or in Fon) that took place between June and August 2021^10^, while also taking into account the writing of the concept note, interviews, translation and transcription of data, and thematic sorting. The fieldwork was conducted by two researchers who assumed responsibility for data transcription and thematic sorting.

Survey populations were selected using the reasoned choice and snowball techniques. Reasoned choice provides that the sample is chosen in a way that represents the studied population as accurately as possible. For this reason, all the groups involved in the vaccination campaign were captured. The sample size was determined by the saturation threshold, i.e., the number of interviewees was stopped when no new codes or themes emerged from the interviews, rather, the same ones started recurring (28, 29). The final sample size was 34, including nine co-morbidity carriers, six health care workers, eleven *traditherapeutes*, and eight people over 60 years old. Particular attention was paid to the gender dimension in the conduct of the interviews to make sure that it was as balanced as possible. Overall, we believe that this mixed-background population could be a good representative sample of the Beninese population. Table 1 reports the characteristics of the interviewees.

**Table 1:** Description of the characteristics of the interviewees.

Average age	48.76 (29–89 years old)
Gender	61.8% Male; 38.2% Female
Category distribution	17.65% Caregivers
	29.41% with co-morbidity
	20.59% over 60 years old
	32.35% traditherapeutes

Finally, the survey data was recorded and then transcribed for the second stage of thematic sorting. When necessary, the transcription was preceded by translation for interviews and passages conducted in local languages (especially French and the Fon dialect). In addition to the interviews conducted, direct observation was chosen as an additional technique for collecting data that could not be captured by speech. The research site was initially identified as Cotonou, but was later expanded to Abomey-Calavi and Seme-Pkodji—two dormitory cities adjoining Cotonou. This choice is justified by the fact that the latter two are affected, in the same way as Cotonou, in terms of the prevalence of COVID-19, in addition to being part of the “red zone” of the pandemic. These choices thus reflected the social urban perception of vaccination, more so because Cotonou and its peripheral towns (Abomey-Calavi and Seme-Pkodji) have 38 vaccination sites out of 78, totaling almost half for the whole country (76 cities).

In the second phase of the project, the results of the field study was analyzed by the University of Warwick team using thematic analysis, triangulation of data collection sources, and content analysis techniques, which was corroborated with a broad literature review. The last (synthetical) phase, in the Hegelian sense, involved combining the results of the first two phases, thus using the framework for proposing strategies and solutions for addressing the identified problems. The framework aims at bringing to public attention the problems identified and interpreted in a pluriprospectic way, and this paper is one of the results and part of the ongoing dissemination strategy.

## Results

**Figure 1:**
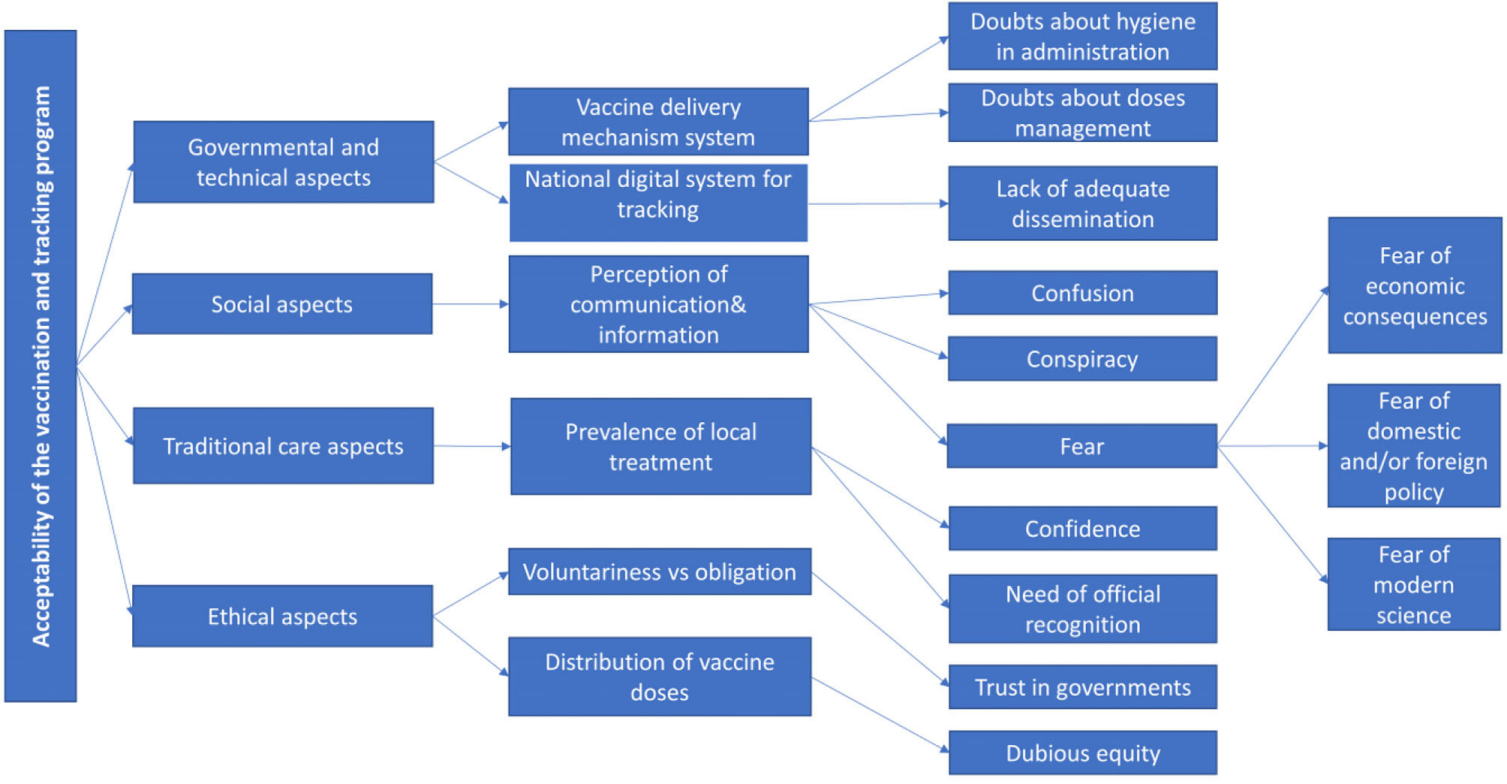
Tree diagram highlighting the main themes surfacing from the interviews.

The conducted interviews revealed many elements worthy of interest related to the social engagement of the Beninese population in the fight against COVID-19. The use of the hermeneutical framework made it possible to identify four macro-areas: (a) governmental and technical aspects; (b) social aspects; (c) traditional care aspects; (d) ethical aspects. It was from these four areas that crucial elements emerged. Figure 1 represents a tree diagram summarizing the main themes pinpointed during the coding of the interviews.

a) Governmental and technical aspects.

The interviewees were requested to give their opinion on the governmental management of the COVID-19 pandemic, including the vaccine delivery mechanism system. The respondents report that the vaccination system followed in Benin is in line with the protocols established by the WHO (30), which registered a good system of vaccine conservation and hygiene in the administration and disposal of waste. However, two accounts were opposed to an overall good opinion of the mechanisms put in place. An interviewee declared his concerns about the hygiene and procedure followed.

“The biomedical devices in place do not always meet the technical needs of the vaccination sites. Here at the National University Hospital Centre, for example, it is not possible to observe the distance in case of turnout. Also, there is no observation room to allow those who feel ill after vaccination to rest (…). The second part I have doubts about is the vaccinator. I don't know if he or she follows the precautions. In principle, after a vaccination, the vaccinator must change gloves before vaccinating another person. But I'm not sure if he respects this standard because they don't have enough gloves. After four or five vaccinations, the vaccinator uses hydro-alcoholic gel. So, they get up from time to time to wash their hands. (Caregiver, male, 54, Cotonou, 09/08/2021).”

Another respondent expressed concerns that temperatures to keep vaccines viable may be subject to fluctuations.

“So, the question is, do we have all the equipment we need to respond? That's the question that came up. I'm already telling you that we don't have a refrigerator in the infirmary and that we put the vaccines in the cooler in the morning until maybe 3 p.m. before vaccinating people, maybe that's a guess, that's my point of view. So, in terms of safety, maybe we're breaking the cold chain a bit. (Health worker, 44 years old, Cotonou, 23/07/2021).”

Most respondents report that they are aware of the system of registration of vaccinations that passes through a national digital system that releases a QR code and is connected to a mobile app^11^. However, some people are unaware of this and report on some procedures that are still paper-based and should be followed by subsequent online data entries.

Ultimately, the Beninese government's handling of the pandemic is perceived in a controversial way. Those who appreciate technical health management are pleased with the government's agenda and attribute its merits to the WHO, while those who are more critical, instead, look favorably on the experiences of other states that have used traditional medicine in the fight against COVID-19 (31) (e.g., the Malagasy president). Moreover, the interviewees show many doubts about the Beninese local government's ability in overcoming the resistance to the vaccination drive due to accusations of corruption (e.g., collusion for economic reasons with the “whites”, poor commitment to the recognition of local traditional medicine, faking the vaccination procedure) and lack of clarity and disinterest in the real involvement of the population.

“Before proceeding to vaccination, the leaders must seek public opinion on the ins and outs of the vaccine and in relation to the rumors that are circulating about the vaccine. Today, Africans have a very high level of awareness and no longer want to submit to the yoke of white imperialism. (…). On the other hand, if it were with our plants and barks, I know that such and such a plant cures such and such a disease, and thus I will be able to submit to the indicated treatment”. (Person over 60, Female, 89, Cotonou, 27/07/2021).

“Now our leaders are being vaccinated to set an example. What is being inoculated into their bodies? We are not doctors to know. What is to be administered to the rest of us as well, we have no idea. Sincerely, they have love for their citizens, let them not be baited by money to feed us.” (Person over 60, Female, 89, Cotonou, 27/07/2021).

“The lack of information means that few people are currently vaccinated. Only intellectuals who have to travel and those who are among the targets listed by the government do so. I can say that the communication around vaccination is not going well. There is no mobilization. Apart from media releases, there is no other form of social mobilization.” (Caregiver, male, 55 years old, Cotonou, 06/07/2021).

b) Social Aspects.

The interviews sought to highlight the position of the population in relation to the COVID-19 pandemic and the reasons that justified them. There is clearly a misconception of the true nature of COVID-19, which gives rise to an overall rejection of the vaccine. Conspiracy theories are very common, and conspiracy against the population is foreseen and explained with the tradition of *zangbeto*, in the local culture the victim par excellence^12^.

“This story talks about the zangbeto that is caught in a mouse trap. In this story of the COVID-19 pandemic, there is something fishy going on; something is being plotted to harm ‘Man'. Something is being hidden from us. We are not being told the truth”. (Person over 60, Female, 85, Cotonou, 28/06/2021).

The emotion that is recorded in a more recurrent way is the *fear* that feeds the denial of COVID-19 and the rejection of the vaccine.

“Some people say that if you get the vaccine, you will die in two years. Maybe that's why people refuse to get vaccinated. Who wants to die? So, people are suspicious. This is the feeling that drives the population; they are afraid. People doubt who is telling the truth and who is not. And so, they decide not to be vaccinated and to remain as they are” (Co-morbidity carrier, Female, 58, Abomey-Calavi, 23/07/2021).

The *confusing information* bombarding the population through communication networks and social media (about post-injection manifestations, the doubts of the government about the age requirements for the vaccination) is the main cause for fear. Social media builds up a real “mobilization strategy” made of WhatsApp messages that are spread by unknown numbers through message chains or forums on the internet. Because this information source is not controlled and/or censored by the authorities, they are considered to be confidential and, therefore, more reliable.

“At the moment, I am not willing to do the vaccination. I am willing to do it if it will protect my health. But, with the rumors on social networks and the alerts on TV channels, and with what is recorded here and there after injections—the illnesses that it causes afterwards, I am not tempted to do it immediately”. (Comorbidity carrier, male, 32, Cotonou, 22/06/2021).

The declared fear can be traced back to three major issues: (a) fear of economic consequences, (b) fear of politics, and (c) fear of modern science.

- *Fear of economic consequences*. This appears to be the major reason for denying the existence of the virus, especially in rural areas that are mostly affected by the restrictions (such as the ban on having markets).

“I have a brother in the village who has a cough. He suffers from a stubborn cough and he is a prominent gentleman in the community who enjoys a certain socio-economic prestige. Eventually, at the local health center, he was found to be a carrier of the COVID-19 virus and had to restrict himself to quarantine requirements. He saw all his activities interrupted, the health center that took care of him is closed to users and the village itself is stigmatized with the adverse effects of poor sales for traders of doughnuts, and especially mustard and Akassa ball, which the locality is known for because of its quality of production.” (Co-morbidity carrier, Female, 55, Cotonou, 29/06/2021).

- *Fear of domestic and/or foreign policy*. As anticipated, the population revealed that they have no trust in the government, based on claims of corruption, and are therefore fearful of any government action. In addition, the fear of politics is also relevant in the international context, and traces its roots to the colonial experience in the past centuries: it is believed that COVID-19 would allow foreign rule to return, thus controlling African populations, or even decimating them in order to limit any anticipated danger that could arise from the increasing population size.

“The anti-COVID-19 vaccine is being introduced in our countries in Africa to shorten the lifespan of the population. The great powers of this world consider that we are overpopulated, there are more people than we need in the world and therefore we have to find a way to reduce the world population size, especially in Africa, to a given threshold. I suppose that we must be 6 billion in the world and here we are at 10 billion! the surplus is thus to be removed”. (Person over 60, Male, 67, Cotonou, 19/07/2021).

- *Fear of modern science*. The population adduces a number of reasons to justify the fear of modern science, coupled with politics. They feel this is another cornerstone of the above-mentioned “anti-African” plot. In particular, some interviewees underline the small number of known COVID-19 infections in Benin and the high temperatures in Africa, from which they deduce that it is a disease that does not particularly develop at such climates and does not particularly affect the Africans' genetics. In addition, with regard to the fight against COVID-19, the population is suspicious of the speed at which vaccines were trialed and believes that this could compromise its effectiveness and safety. In this regard, other respondents reinforce the hypothesis that it is a political-economic expedient because they are dubious about the reasons for the speed behind vaccine production and spread in spite of the fact that other more common diseases in Africa are still waiting for a cure (e.g., malaria or AIDS). This also brings up the suspicion that the African population is being used as “guinea pigs” to test certain types of vaccines. All these reasons reinforce the general rejection of not only the COVID-19 vaccine, but also other vaccines that were accepted in the past.

“COVID-19 is not in our country. It is the white people who brought it”. (Tradithérapeute, Male, 35, Cotonou, 16/06/2021).

“The whites do not like blacks. With the number of deaths caused by COVID-19, they do not take action to save themselves and they want to save us. This way of acting should make black people think. AIDS has been around for years without a vaccine, and it's COVID-19 that they have found a vaccine for. I don't want this vaccine!”. (*Tradithérapeute*, Male, 35, Cotonou, 16/06/2021).

“In the face of every disease, Africans are used as test drivers. They use us as test subjects to improve their products. It is true that the whites have already started taking the vaccine. But the drugs they use are different from the ones they send to us in Africa. The quality of the pharmaceutical products is not the same. That's why our Heads of State go abroad for treatment when they are ill”. (*Tradithérapeute*, Female, 33, Cotonou, 16/06/2021).

“As a result, I have had to dismiss the polio vaccinators who go to households door-to-door because we don't really know any more if they are polio vaccinators or if it is the COVID-19 vaccine”. (*Tradithérapeute*, male, 35 years old, Cotonou, 16/06/2021).

These convictions nourishing the social perception of the ongoing pandemic are extremely acute in the peri-urban and rural areas where there is a widespread belief that the virus is fictitious. Also, the distance from the vaccination mechanism, which is concentrated in urban areas, and the government's disregard in respect of the control of compliance with safety measures contribute to these beliefs.

“The disease is not in our villages. The villagers ask us to be careful. They ask us not to come and contaminate them. Because the disease is not in the village. It's all fabrications when they tell us that there are cases of COVID-19 in the villages”. (*Tradithérapeute*, male, 34, Cotonou, 16/06/2021).

“In the villages, compliance with these rules was lower. But in our urban areas, the police will arrest you if you don't respect the barriers”. (*Tradithérapeute*, Male, 34, Cotonou, 16/06/2021).

“It is in Cotonou that we can say that people are more or less aware of COVID-19 restrictions, but when they leave Cotonou, it is not so evident. (…) In Cotonou, you can't go into a pharmacy without a mask, but in Parakou, for example, you can go into a pharmacy without a mask, which reduces the motivation to get vaccinated”. (Comorbidity carrier, male, 43 years old, Sémé-Podji, 26/07/2021).

c) Traditional care aspects.

In Benin, there exists a rich context of traditional medicine based on the use of plants for self-medication and the multiplicity of traditional healers (i.e., *traditherapeutes*), many of whom are recognized by the health care system, while others are considered charlatans. The main problem of traditional medicine is that there are no scientific studies that establish doses and confirm full alternatives to modern treatments. Nevertheless, there is a great engagement of the population (including medical doctors) in this type of treatment. The interviewees reveal the strong belief that traditional medicine is a valuable preventive prophylaxis tool against COVID-19, and think it is better than any other modern treatment.

“We have strengths in traditional medicine that can help us prevent and cure even COVID. These are the recipes and I believe in them. We are told that COVID-19 is like the flu that chloroquine can cure. But we also have drugs that play the role of chloroquine and Azithromycin. So the problem is solved traditionally.” (Caregiver, male, 54, Cotonou, 09/08/2021).

“Do you see in the West, despite the development in their health systems, that their populations have suffered a hecatomb? Have you not asked yourself why not here? It is precisely because of our food and pharmacological habits based on plants! These people, we must not give in to ignorance and simply follow them. They will simply kill us. No, in Africa we have everything we need to cure all kinds of diseases”. (*Tradithérapeute*, Male, 66, Abomey-Calavi, 23/07/2021).

The respondents are aware that studies of traditional medicines require time and investment of money. Respondents report that some local products are licensed, while others have been banned. This gives rise to the fear of government repercussions against the traditional healers. In fact, based on some reports, the government tried to involve traditional healers in the management of the pandemic by creating a group of experts in traditional medicine. This however did not give them the opportunity to take initiatives. In reality, local governments are acting very cautiously with respect to local medical traditions, fearing international repercussions (as in the case of Madagascar, where the president sponsored local treatment against COVID-19, but later withdrew the regulations following pressures from the international community).

“The problem of complementarity is primarily a political and geostrategic issue of the great powers. To date, no blacks have been allowed in the circle of researchers conducting research on COVID-19. Without protection, we cannot exhibit our products against COVID-19 because we will be fought. The case of the Malagasy President is an example. The second example is that of Dr. Agon Valentin. Burkina Faso had accepted his product, API-VIRIL, as a remedy against COVID-19. However, when the ‘Whites' got involved in the dance, his product was banned from use against COVID-19, and was withdrawn from the Burkinabe market and banned from sale in his country, Benin.” (*Tradithérapeute*, Female, 37, Cotonou, 16/06/2021).

“In fact, there are many problems at this level. First of all, there is the problem of the non-complementarity of the biomedical and endogenous offer. Moreover, the promising endogenous physicians have difficulty in getting together to produce quality medicines. The quest for individual recognition leads them to evolve in a scattered manner. As an example, to face the COVID-19 pandemic, the Beninese government has set up a committee of experts. This committee met with the traditional medicine actors that we are. It asked us to organize ourselves to make proposals for a remedy against the pandemic. Instead of uniting to produce something of quality, each healer went to the committee of experts with their product. It was so disorganized that the expert committee has not made a decision to this day yet. So, it's an internal problem for us.” (*Tradithérapeute*, Male, 34, Cotonou, 16/06/2021).

Fear recurs as the dominant theme in the interviews, and one of the remedies to fear is considered to be traditional medicine. In fact, some respondents report that before vaccination against COVID-19, some people had undergone a spiritual purification and made specific sacrifices to local deities, or consulted the oracle of local geomancy. This represents another side of traditional medicine, not necessarily related to herbal treatments.

“There are some who are vaccinated and have not had any effects. But when I approached them, they told me that they prepared themselves psychologically, medically and spiritually by giving themselves to God to become immune to COVID-19. They too were afraid and took steps to overcome the fear. A few days before taking their doses, they took chloroquine and azithromycin to fight against the virus and paracetamol to prevent fever, as is done for children during vaccination. They also prayed a lot and did not want their husbands and wives to follow suit. The others did it first to see what it would do”. (Caregiver, woman, 45, Cotonou, 12/08/2021).

“There is also that deity ‘Sapkata^13^'. Let us not forget it. It is ‘Sapkata' who governs all diseases. If one makes the required sacrifices to her, she protects and prevents the population from falling ill”. (*Tradithérapeute*, Male, 34, Cotonou, 16/06/2021).

“All illness comes from a spiritual imbalance that negatively impacts the physical condition. Therefore, if we can regulate all spiritual imbalances, no one will fall sick because illness is a spirit. It is enough to make Soudjo conjure the spirits responsible for the disease so that they do not come into our environment.” (*Tradithérapeute*, Male, 34, Cotonou, 16/06/2021).

“Moreover, we had consulted the Fa oracle about the origin of COVID-19. […] The Fâ was clear and precise. He even said that young people will not be victims of this disease. Following these revelations, we made sacrifices. Only charlatans paid money to make the sacrifice against COVID-19. We should then do the ritual called tokplopklo. But due to lack of funds, this has not yet been done. The tokplopklo^14^ is done by several people. The government itself has to give money. But it refuses to provide the necessary means. This is proof that our leaders do not love their country.” (*Tradithérapeute*, Male, 66, Abomey-Calavi, 23/07/2021).

“In addition, we have other methods to send the disease away from our lives. These include taking adjanouhlahoun and zozoman. When you take a bath with the potion of these two leaves, any disease stays away for a good period of time.” (*Tradithérapeute*, Male, 34, Cotonou, 16/06/2021).

Ultimately, it emerges that the population feels more represented by traditional medicine than by modern medicine and would very much appreciate the formalization of the former. It is also evident that an official involvement of traditional medicine in the fight against the pandemic would lead to a stronger popular engagement.

“But I think the authorities need to approach our traditional healers to learn about the recipes they use, test them scientifically before accepting or rejecting their use. If not, we will lose. Besides, we are used to losing. Because our old people are dying every day with their recipes. The best strategy would be to accept these traditional plants, study them scientifically and make them available to the population. Researchers are there for that. Our governments must change their positions. They know full well that traditional medicine is full of qualities. So we should not categorically refuse without experimenting.” (Caregiver, Male, 55, Cotonou, 06/07/2021).

d) Ethical aspects.

In Benin, vaccination is voluntary. In the first phase, it was limited to some categories (i.e., health professionals, people with serious comorbidities, over 60 years), but now, it is open to people aged 18+. A specific section of the interviews was dedicated to asking how the population considered the theme of mandatory health treatments, and if the vaccine against COVID-19 was perceived as an obligation. We discovered that many health care workers were also opposed to vaccination, although they were not obliged to get it done at that time.

“So far, nothing! I thought that if someone doesn't get vaccinated, they won't be able to do their job, that they will be fired, but so far there is no such pressure. Vaccination is not mandatory for health care workers. But if it were compulsory, I would make one condition: I have a medical certificate describing my state of health and I would write to the authorities that it's against my will and that I'm getting vaccinated to save my job.” (Caregiver, female, 45, Cotonou, 12/08/2021).

As regards the COVID-19 passport, the population is opposed to it and considers it as a form of oppression.

“It is wrong to talk about the COVID-19 vaccination passport. But we are not going to let it happen. However, it's a pity”. (*Tradithérapeute*, male, 34, Cotonou, 16/06/2021).

Finally, the interviewees underlined the lack of equitability in the distribution of vaccine doses: the sharp difference between areas of the world prevents a global community vision.

“What did not work was the international community. It does not have that kind of solidarity. If everyone is affected, everyone should benefit equally from the vaccine doses. But today we see that the Westerners give priority to themselves first, and then send us a few drops. That didn't work. The mobilization of resources has not worked. I'm sure that the resources that countries have received and mobilized have not allowed them to do much. (Co-morbidity carrier, male, 43 years old, Cotonou).

## Discussion

The fight against COVID-19 will be successful only if it will build on a complete engagement of the population. As shown in some of the reported interviews, the latter is strictly connected to many sociocultural aspects. The first aspect is the net gap between the population and politics, which often relies on contradictory and unclear communication strategies for the population, thereby fomenting conspiracy theories and negationism. Overall, it seems that local policy is geared more toward complying with international standards rather than to assessing and tackling what is happening in the country.

Firstly, Benin is carefully trying to follow the WHO protocols of conservation, administration and disposal of waste, but does not deal with the very serious risks that could result from blackouts or unstable electrical supply to the refrigerators containing vaccines. The cold chain (i.e., cold storage during the delivery and storage at the provider's facility up until the administration to the patient) is required by some vaccines to maintain their potency and effectiveness. This may be very difficult to maintain in a LRS such as Benin, which has an electrification rate of 43% and a quality of electricity rated as 2.06/7 (as a comparison, Italy has a 100% electrification rate, and a quality of 5.91/7). The quality of the electricity is compromised by frequent blackouts, sags and swells, and the frequent unavailability/breakdown of generators, as also reported in previous studies (32, 33). The more general concern is that for politics, economics, and in the present case, also health, there are established International Standards, which although have the benefit of standardizing practices and facilitating trade between countries, are in fact limited by not considering the variety of specific contexts and situations. The alleged universality of standards is often generic and risky because, as in the case of the storage temperature of vaccines, it could call into question the effectiveness and safety of health technologies and, consequently, the health and rights of patients.

The second evidence of the policy's distance from the population is that there is lower control over distancing measures and spread of vaccination and its tracking mechanism, particularly as observed in the Beninese rural areas. This significantly affects popular engagement, which is clearly more limited in those areas and feeds the denial. Emerging from the interviews are many reasons given for the denial of COVID-19 and vaccine refusal, including alleged environmental or genetic reasons and the so-called “neo-colonialism”. In fact, the population believes that more than as a health emergency, this pandemic could be better described as a political alarmism with economic aims. This latter motivation finds its roots in the centuries of colonialism that still persist in the memory of the population so vividly; mainly that some people believe and spread conspiracy theories against the African population. One of the most interesting supported theories is that the low prevalence of the disease does not justify preventive prophylaxis compared to many other more common diseases with much higher mortality rates that have long deserved the search for a vaccine or cure. It would seem that a common belief is that the world wants to vaccinate Africa to protect itself, and that if the pandemic is not eradicated globally, there will always be the danger of its comeback, possibly in the form of even newer variants (two of the most recent ones were already firstly identified in Africa). On closer inspection however, it is clear from the interviews that local people do not want to fight a battle which is being disputed elsewhere, i.e., a disease that is not of concern in their own country. Even more so, local people are suspicious and angry about the underlying faulty mechanism behind it all (e.g., the COVID-19 vaccine discovery and testing in such short times), which did not consider as priority other epidemics or diseases mainly affecting Africa.

For these reasons, one of the most obvious lessons learnt from the pandemic is the urgent need to reflect once again on the *priorities*, that is, the ethical criteria that drive the progress of science, globally. Generally, the interviews highlighted the belief that third parties (international and/or local politicians, pharmaceutical companies, “whites”) for various reasons (economic, political, and experimental) are conspiring against the population (i.e., *zangbeto* metaphor). These fears reveal once again a clear gap between the people and the politicians, creating a situation where the populace fear the behavior of elected individuals who, in a democratic government, should represent them. This evident crisis of democracy motivates the attachment to popular beliefs concerning political charlatans, or those spread *via* social media, or by conventional wisdom.

As mentioned earlier, the most recurrent feeling interwoven with the interviews is that of fear. Fear has always been a philosophical, as well as a psychological concept on which many contemporary theorists have developed important reflections: from the heuristics of fear, theorized by Hans Jonas, for which profiling a terrifying future picture of the consequences of one's actions could favor the “principle of responsibility” (34), capable of promoting responsible actions to protect future generations, up to the reflections of the American philosopher Martha Nussbaum, a theorist of the social democracy based on skills and capabilities, who also modernly argues about the “monarchy of fear” (35), according to which fear is no longer part of an emotional dimension of the private sphere, but is part of a wider public sphere, affecting mass behaviors, just as it happened during the so-called “COVID-19 era”.

In the case of Benin, the fear of oppression, linked to historical resentment toward the West, has led to the habit of identifying external culprits, thus fueling undemocratic attitudes. In this respect, Nussbaum argues that the wrath-blame mechanism is typical of fear. On closer inspection, however, fear is a primitive, narcissistic, and asocial emotion that is still causing more serious consequences in some countries than the spread of the virus itself. The fear of the vaccine is not a new thing for animist cultures. According to general belief, inoculation introduces pathogens that cause “something sick and evil”, a situation which most would interpret in a spiritual sense (36). Currently, the traditional fear of the vaccine is intensified, evidenced by the refusal of the COVID-19 vaccine, with the implication that people might begin to reject vaccinations for other diseases too. Nonetheless, this should not lead us to think that the population rejects the COVID-19 vaccine for religious reasons, though it is a phenomenon common to several religions, which verified the compliance of vaccine serums with their religious dietary requirements. Islam and Judaism, for example, prohibit the intake of pork and its derivatives, while the Hindu religion prohibits the intake of cattle derivatives (37).

On the contrary, in Benin, the extraordinary tradition of traditional medicine, cloaked in its dual spiritual and phyto-therapeutic nature, is readily available for searching causes and remedies related to the pandemic. Unfortunately, the government appears to distance itself from what represents very valuable local knowledge, even for primary health care. In fact, not only has the government refused to do an in-depth study of traditional medicines by assessing their effects and establishing their correct dosages, but also withdrew some herbal remedies that were either being used against COVID-19 or for strengthening the immune system from the open market (e.g., artemisia). This disposition again shows the government's detachment from a confused and frightened population left to act alone and in secret. Certainly, in times of emergency, it is expensive and tedious to undertake studies parallel to official ones on alternative remedies to COVID-19, but it is also true that traditional medicine experts could support research and, at the same time, represent a reliable reference for the community to facilitate the engagement of the population (38). Notably, an attempt to officially appoint a group of traditional medicine experts to provide a solution to COVID-19 was made, but was unsuccessful due to a lack of organization and capacity for initiative.

Overall, as we have seen, there are many reasons behind the rejection of the COVID-19 vaccine. Among all, social engagement should be considered a priority. When considering feedback from the interviews, it would seem that social engagement poses a greater challenge than the much-debated issue of the unfairness in the distribution of vaccines. Even if there were the availability of free and universal vaccination coverage, the popular rejection of vaccination would yet remain an obstacle to global health.

The aforementioned document of the Beninese government announcing the opening of the vaccination campaign states “Vaccination against COVID-19 is effective, safe, voluntary and free. Get vaccinated to protect yourself and others”^15^. Voluntariness corresponds to the ethical principle of *autonomy* that ascribes to the individual the balance between *risks* and *benefits*. According to the interviews, it would seem that the population is particularly frightened by the risks (including post-injection complications, scams made by governments, and the fear of dying). The benefit of vaccination, that is, individual and mass immunization, is underestimated because, from the personal point of view expressed by most interviewees, it is believed that other diseases are more widespread in those places (39) and there is no urgent need for this specific vaccine. The prospect of achieving global health should instead be mindful of historical heritage since the local belief is that the population is being used as “guinea pigs” to test health solutions for the sake of others.

Since people would be naturally more inclined to choose personal interests over collective ones, it is highly imperative that they are properly educated in making the right choices, especially as this improves the fight against possible pandemics and their behavior in times of emergency. Moreover, it is not possible to predict whether in the future the countries which are now relatively unaffected by the pandemic will witness an increase in infections, resulting in even more disastrous technical and medical consequences. This is why the Beninese government has not followed the risk-benefit balance, but rather, the *precautionary principle* in promoting mass vaccination of the population. However, considering the low record of population engagement in the fight against COVID-19, if the Beninese government wants to pursue the immunization goal, the question to ask is; should it give up the *autonomy principle*? From a theoretical point of view, it should be pointed out here that the *principle of autonomy*, which is at the basis of informed consent and dissent, is exercised when a competent individual, i.e., someone made clearly aware of the health framework, makes a decision in accordance with their moral perspective. However, in the present case of COVID-19, and taking infodemics into consideration, it would seem that the lucid and genuine self-determination of the individual has been seriously compromised. This is well linked to the ethical principle of *trust*, which is not built on authority, but on the relationship, involvement and genuine inclusion of the population in scientific and political healthcare management. Trust should be built with clarity and honesty, and with sufficient explanations on any eventual limit or uncertainty of science (40). As of now, the emergence of rapid publication of information has called into question the accuracy of the information being circulated (41, 42). This phenomenon can be stopped by appealing first to scientists and researchers to be responsible and conscientious in their work, before even reaching out to those responsible for the circulation of information on communication networks and/or social media.

One of the first steps to clearer management of the pandemic would be to follow the infections more strictly with more capillary testing and digital tracking mechanisms (43). Many countries have followed this path, starting from the “immunity passport” (44) (which certifies whether a person has received the COVID-19 vaccine or has recently tested negative^16^). These passports permit holders to return to some of their normal activities, such as traveling more freely and returning to work (45). An immunity certification program could complement population or community-based strategies to ease restrictive measures, as well as secure societal and individual freedom and wellbeing.

Leaving aside the non-negligible issue of limited access to technologies (such as smartphones) and internet coverage, which could be the first obstacle to full deployment of the procedure, Benin has put in place an online portal hosting an app that can be used by vaccinated people to download and show a QR code as a proof of their status. Moreover, due to the limited number of people who accepted the vaccine, the government is prevented from limiting access to certain activities to only the vaccinated people, as well as from returning to severe containment measures such as lockdowns. Certainly, the immunity certification should never be used as the main strategy for reducing the effects of the COVID-19 pandemic, but could be used as a part of a plan that provides for a decrease in the number of people subject to highly restrictive measures (46, 47). As it is now, the immunity certification is an intermediate management strategy between mere recommendation and obligation, with related sanitation measures for whistleblowers—a balance between autonomy and public health.

The trust of the population must be built based on the recognition of local actors as community leaders, or even *traditherapeutes* that can facilitate popular engagement in the vaccination program (48, 49). This is related to the ethical principle of *equal moral respect*, which is particularly interesting in relation to our study. This principle provides that “the interests of all be taken into equal account” and also requires “being sensitive to cultural diversity and plurality”, which in turn requires a willingness to engage in dialogue and deliberation in terms of equal standing and recognition. In practice, this means ensuring that potential participants are empowered to reach their own decision regarding whether they would like to participate, ensuring that consent is sought in a culturally appropriate manner, and addressing participants' perspectives or concerns about the research, including information about how their data and samples will be handled, and so forth” (7). This could be achieved by an adequate study of local medicines and traditional therapeutic practices that can go hand in hand with the development of modern medicine.

This brings us back to the ethical principle of *equity*, which is not only related to a fair distribution of resources, but also to a consideration of specific popular needs: “for people to be treated equitably, they should be able to exert at least some influence over the decision-making process as well as the decision itself, i.e., procedural fairness”^17^. Beyond this, including individuals and communities means maximizing the social value of science and politics (50). All points mentioned beforehand respond to the ethical principle of *solidarity* (51), which encompasses the respect of the pluralism of cultures and human rights. In the present case, human rights pertaining to health initiatives are individual and at the same time global, and go hand in hand with the right to enjoy the benefits of science and its results (in this case COVID-19 vaccines), all in respect of an individual's right to cultural identity. Furthermore, the latter point should be related to the right to having an adequate education, which puts a person in a position to clearly understand what is happening so as to exercise autonomous choices responsibly.

## Conclusion

The world shares a collective responsibility in fighting the present pandemic; therefore, the reluctance of many local populations to accept the vaccine or their hesitancy toward tracking COVID-19 cases highlighted the urgent need for a study on social engagement in the fight against COVID-19. In particular, this paper shows the example of Benin, a Sub-Saharan country characterized by multiple endogen differences and a rich cultural tradition of animism and traditional medicine. With this interdisciplinary study, we explored the popular perspectives and beliefs on the pandemic from a sociological, ethical, political and scientific point of view. In order to be able to use a multi-level angle view and engage in multi-level discussions, an interdisciplinary framework was used. This framework, which makes use of multiple methodologies, allows schematization within a broad question and a number of issues, and provides tools to facilitate discussions at different levels. Moving away from a top-down approach and favoring a bottom-up one is considered an important way for multi-directional learning and for shaping an effective response informed by values.

The outputs emerging from the interviews, however, suggest a clear separation between the public and private sphere, and the distance of politics from the population, especially in peri-urban and rural areas, where negative perceptions and feelings of being ostracized prevail. The objective of this framework is to bring to public attention particularistic perspectives and how these suffer from the imposition of universal standards and procedures, i.e., in this case, the protocols of vaccine administration and tracking. The framework proposes flexible solutions to address these gaps (e.g., social mobilization strategy through the involvement of local medical expertise) because social engagement is vital to engage an effective immunization process. Current researchers should understand which modalities of public engagement are most effective. This study illustrates the urgent need for connecting clinical practice, public health, and social policy decision tables with broader community concerns, while also relying on bioethics. For the future, one of the lessons that should remain from the pandemic is that public perception should be considered a priority in the future management of health care emergencies, thus delivering the stamp of a “job well done” to posterity. One might argue that one of the limitations of this study is the limited sample size, which may not be representative of the whole Beninese populations. However, both the fact that our sample includes people with different cultural and socio-economic backgrounds, and our use of the aforementioned saturation threshold to capture all the possible themes, make this limitation negligible. Nonetheless, further comparative ethnography studies could focus on other populations of Sub-Saharan Africa to see whether similar beliefs and perceptions are shared within wider communities.

## Data Availability Statement

The original contributions presented in the study are included in the article/Supplementary Material, further inquiries can be directed to the corresponding author.

## Ethics Statement

The studies involving human participants were reviewed and approved by Ethical Committee of the University of Abomey-Calavi. The patients/participants provided their written informed consent to participate in this study.

## Author Contributions

AM wrote the main manuscript text. MV and AG conducted the field study. AM, DP, and IO analyzed and reviewed the results. AM, DP, and MV prepared all the Supplementary Materials. AM, LP, and RH contributed to the conceptualization. IO and DP involved in supervision and editing. LP involved in funding acquisition. All authors contributed to the article and approved the submitted version.

## Funding

This research was funded in part by the University of Warwick with two Warwick Impact Found Grants supported by the EPSRC Impact Accelerator Award (EP/K503848/1 and EP/R511808/1).

## Conflict of Interest

The authors declare that the research was conducted in the absence of any commercial or financial relationships that could be construed as a potential conflict of interest.

## Publisher's Note

All claims expressed in this article are solely those of the authors and do not necessarily represent those of their affiliated organizations, or those of the publisher, the editors and the reviewers. Any product that may be evaluated in this article, or claim that may be made by its manufacturer, is not guaranteed or endorsed by the publisher.

## Footnotes

^1^The Pfizer/BioNTech Comirnaty, 31 December 2020. The SII/COVISHIELD and AstraZeneca/AZD1222 vaccines, 16 February 2021. The Janssen/Ad26.COV 2.S developed by Johnson & Johnson, 12 March 2021. The Moderna COVID-19 vaccine (mRNA 1273), 30 April 2021.The Sinopharm COVID-19 vaccine, 7 May 2021. The Sinovac-CoronaVac, 1 June 2021. The Bharat Biotech BBV152 COVAXIN vaccine, 3 November 2021.

^2^ https://covid19.who.int/

^3^ https://covid19.who.int/table

^4^https://ourworldindata.org/covid-vaccinations globally

^5^ https://www.who.int/initiatives/act-accelerator/covax

^6^ https://www.who.int/news/item/23-09-2020-managing-the-covid-19-infodemic-promoting-healthy-behaviours-and-mitigating-the-harm-from-misinformation-and-disinformation

^7^ https://unsdg.un.org/latest/stories/benin-national-covid-19-vaccination-campaign-has-launched

^8^ https://www.gouv.bj/coronavirus/#documents

^9^ https://covid19.who.int/region/afro/country/bj

^10^The study received full ethical approval from the University of Abomey-Calavi Comitè d'Ethique de la Recherche CER-ISBA (No. 131).

^11^ https://www.afro.who.int/news/benin-goes-digital-offensive-against-covid-19

^12^The zangbeto is the composition of two phonemes: “zan” meaning “night” and “gbeto” meaning “hunter”, i.e., night hunter. The zangbeto is, from a social point of view, the traditional night watchman and a secret society of masks in southern Benin. When he goes out, the zangbeto, being masked, is accompanied by zanvi (followers of the zangbeto) and if he is caught by a mouse trap, it means that he is a victim of a trap. Otherwise, the people are victims of a conspiracy.

^13^Sapkata is a deity of southern Benin, of Yoruba origin, reputed to be one of the dominant figures of the Vodun pantheon. It is considered as the regent on earth and the God of eruptive diseases. It is usually the beneficiary of expiatory rites in cases of epidemic.

^14^Soudjo and Tokplopklo are expiatory rites literally known as baths of purification for the community life to ostracize the evil from the socio-linguistic group.

^15^ https://www.gouv.bj/coronavirus/#documents

^16^Although this second option gives very limited freedom as the obtained pass lasts only a few days.

^17^Ivi, p. 129.
