# Supplementary material for: Social Engagement in the Fight Against COVID-19 in the Urban and Peri-Urban Areas of Cotonou (Benin, Sub-saharan Africa): Acceptability of the Vaccination and Tracking Program
Source: Front Med (Lausanne). 2022 Jun 3;9:857890. doi: 10.3389/fmed.2022.857890 (PMC9203714; doi:10.3389/fmed.2022.857890)
Supplement: Supplementary file 1 [file Table_1.DOCX]

Supplementary Material

**Interview guide**

1. **To Caregivers, people of 60 years old and people with co-morbidities**

**Introductory word**

Hello Mr / Mrs, thank you for the time you have been kind enough to grant us. My name is …, research assistant in health anthropology at the University of Abomey-Calavi. We are researching the engagement of the population in your area in the fight against Covid-19, with respect to the acceptability of the vaccine and tracking. We have identified you as a resource person who can provide us with reliable information in order to better understand and analyse the facts. This interview will take 45 minutes to 1 hour and 30 minutes to complete.

We reassure you that this survey is completely anonymous. All the data that you will provide to the interviewer during the interview will be kept strictly confidential. Your name and other identifiable information will not be disclosed nor mentioned anywhere.

**Context of the interview**

-Date and place

- Start time and End time

- Circumstance of the interview and other useful information

**I. Identification of the interviewee**

- Surname and first names (optional)

-Age

- Gender

- Educational level

-Marital status

- Socio-professional activity (ies)

- Sociolinguistic group

- Religious affiliation

**II. Social history of biomedical misfortunes and epidemics**

- Acute diseases known and / or the locality of origin: aetiology and signs, people affected and mode of transmission, name and therapeutic routes

- Known chronic diseases and / or locality: aetiology and signs, people affected and mode of transmission, name and therapeutic routes

- Known epidemics and / or the locality of origin: aetiology and signs, people affected and mode of transmission, name and therapeutic routes

**III. Popular representations relating to COVID-19 and the timeliness of the vaccine**

- COVID-19 pandemic: etiology, semiology, modes of transmission, risks and associated lethality

- Anti-pandemic control: exposed subjects, barrier gestures and mobility restrictions, screening

- Prevalence of epidemiological trends: evolution of cases and biomedical management, wide variability depending on the region.

- Anti-COVID-19 vaccines developed and introduced in Africa, especially in Benin

- Vaccine efficacy: to justify the point of view

- Sources of information – satisfaction in the scientific/political communication

- Populations predisposed to be vaccinated or not: to justify the point of view

- Population preferences of the type of the vaccine available in the world.

- Reasons to refuse vaccine (fear, misinformation…)

- Preventive alternatives to the fight against COVID-19 to the vaccine.

- Popular perception of the allocation of resources (what are the categories of population that should receive the vaccine?)

-Differences among rural population and citizen perception of vaccine.

- perception of the vaccine as an obligation or voluntariness

- perception of the Covid vaccination passport

-What happens to healthcare workers who refuse to get a vaccination? Are they still allowed to work in the hospital?

- Considered the limited extent of Covid-19 diffusion in Benin, investigate how the population perceive the opportunity of the vaccine (risk-benefit or equality)

**IV. Bio-sanitary vaccination system in place**

- Organizational aspects in place:

- Imposition of the COVAX initiative on independent States: modality of eligibility of priority target populations, imposition of the source of supply and quantity of acquired doses,

- Impossibility for undefined priority populations wishing to be vaccinated.

- Technical aspects: compliance with standards

o Logistics of inputs and equipment (refrigerator availability/ lack of energy/ waste disposal)

o Equipment maintenance

o Problem of the cold chain

o Qualified personnel

o etc.

o distance of vaccination sites from rural areas (this discourage inhabitant of the rural area?)

o adequate information for the informed consent

o relation among first and second dose (side effects, people who give up

o vaccine tracking or side effect app/computerized system to reach people to vaccine and vaccinated (if yes, this give the impression of monitoring and control everyday life

**V. Social mobilization strategy to generate community involvement**

- Populations normally required for administration of the vaccine: to be justified

- Categories of populations (03) retained by the vaccination plan: to justify the point of view in relation to the criteria in question

- Means deployed to ensure their involvement in the vaccination process:

o Ex ante consultation frameworks in decision-making

o Process and result

- Choice of vaccination sites

- Choice of healthcare personnel positioned

- The vaccine administration protocol (three phases) and compliance

- Resources mobilized for a disease proven to be the 32nd cause of death for African countries,

**VI. Popular therapeutic norms**

- Biomedical therapeutic alternatives in the face of COVID-19

o Offers: for which audience and for what purpose

o Governmental position related to the traditional treatments anti-Covid as alternative to the vaccines

o Use of local therapies: why such a choice …….

o The problem of the non-complementarity of the biomedical supply and the endogenous supply.

Final word!

1. **To Traditional therapists**

**Introductory word**

Hello Mr / Mrs, thank you for the time you have been kind enough to grant us. My name is …, research assistant in health anthropology at the University of Abomey-Calavi. We are researching the engagement of the population in your area in the fight against Covid-19, with respect to the acceptability of the vaccine and tracking. We have identified you as a resource person who can provide us with reliable information in order to better understand and analyse the facts. This interview will take 45 minutes to 1 hour and 30 minutes to complete.

We reassure you that this survey is completely anonymous. All the data that you will provide to the interviewer during the interview will be kept strictly confidential. Your name and other identifiable information will not be disclosed nor mentioned anywhere.

**Context of the interview**

-Date and place

- Start time and End time

- Circumstance of the interview and other useful information

**I. Identification of the interviewee**

- Surname and first names (optional)

-Age

- Gender

- Educational level

-Marital status

- Socio-professional activity (ies)

- Sociolinguistic group

- Religious affiliation

# II. Social aspects: social factors that interfere with vaccine acceptability

- What do you think of Covid-19 and the Covid-19 vaccination?
- Have you been vaccinated?
- If so, why?
- If not, why not?

**III. Traditional care aspects**:

- Are there any traditional treatments used by the populations to prevent COVID-19?

o If yes, which ones o If not, why?

- What do you think of these treatments (in terms of effectiveness, quality and safety)?
- What do the national authorities think about these treatments?
- What do people think about these treatments?

# IV. Ethical aspects

- What do you think of the promotion of vaccines by governments instead of the local therapies you propose?
- What do you think of the vaccination procedure required for entry into and exit from Benin?
- What do you think about the cost of vaccinations being charged to passengers?

Final word!
